# Synthesis and Characterization of Cationic Glycidyl-Based Poly(aminoester)-Folic Acid Targeting Conjugates and Study on Gene Delivery

**DOI:** 10.3390/molecules17089056

**Published:** 2012-07-30

**Authors:** Pai Feng Tsai, Wei Yang Chang, Yu Che Hsiao, Kuo Jung Li, Min Da Shau

**Affiliations:** 1 Department of Occupational Safety and Hygiene, Chia-Nan University of Pharmacy and Science, 60 Erh-Jen Rd., Sec. 1, Jen-Te, Taiwan; Email: paifeng@mail.chna.edu.tw; 2 Department of Biotechnology, Chia-Nan University of Pharmacy and Science, 60 Erh-Jen Rd., Sec. 1, Jen-Te, Taiwan; Email: oz.j.w.y@hotmail.com (W.Y.C.); dragonli@mail.chna.edu.tw (K.J.L.); 3 Department of Materials Science and Engineering, University of Tennessee, Knoxville, TN 37996, USA; Email: k7310162002@gmail.com

**Keywords:** poly(aminoester), folic acid, transfection, cytotoxicity

## Abstract

A new poly(aminoester) (EPAE-FA) containing folic acid and amino groups in the backbone and side chain was synthesized. EPAE-FA self-assembled readily with the plasmid DNA (pCMV-βgal) in HEPES buffer and was characterized by dynamic light scattering, zeta potential, fluorescence images, and XTT cell viability assays. To evaluate the transfection effect of graft ratio of FA on the EPAE system, EPAE-FA polymers with two different graft ratios (EPAE-FA12k and EPAE-FA14k) were also prepared. This study found that all EPAE-FA polymers were able to bind plasmid DNA and yielded positively charged complexes with nano-sized particles (<200 nm). To assess the transfection efficiency mediated by EPAE and EPAE-FA polymers, we performed *in vitro* transfection activity assays using FR-negative (COS-7) and FR-positive (HeLa) cells. The EPAE-FA12k/DNA and EPAE-FA14k/DNA complexes were able to transfect HeLa cell *in vitro* with higher transfection efficiency than PEI25k/DNA at the similar weight ratio. These results demonstrated that the introduction of FA into EPAE system had a significant effect on transferring ability for FR-positive cells (HeLa). Examination of the cytotoxicity of PEI25k and EPAE-FA system revealed that EPAE-FA system had lower cytotoxicity. In this paper, EPAE-FA seemed to be a novel cationic poly(aminoester) for gene delivery and an interesting candidate for further study.

## 1. Introduction

Nonviral gene delivery systems based on complexes of condensed DNA with polycations have attracted great attention for their pharmaceutical applications in treating chronic diseases and genetic disorders [1,2]. Cationic polymers and lipids can potentially provide many advantages because of easier control of their molecular compositions, simplified methodolgy, low immunogenicity and cytotoxicity, and high gene loading [3,4]. Cationic polymers not only condense DNA into nanoparticle smalls enough to enter cell through endocytosis, but also protect negatively charged strands of DNA from nuclease degradation. Additionally, cationic polymers can provide a pH-buffering ability allowing them to behave as a “proton sponges” which could assist in the escape of polycation/DNA complexes from lysosomes and improve the transfection efficiency [5,6]. It has been demonstrated that the presence of polymers mediates transfection, but they were also associated with a considerable degree of cytotoxicity. The polymers containing amino groups such as poly-L-lysine (PLL) [7], poly(2-(dimethylamino)ethyl methacrylate (PDMAEMA) [8], polyethylenimine (PEI) [6], and polyamidoamine [9] have been proposed as carriers for genetic material because they readily form complexes with DNA. However, these polymers are nonbiodegradable materials. Consequently, there have been great efforts to synthesize biodegradable polymers that can be used as gene vectors with low cytotoxicity. A number of reported biodegradable gene carriers include poly(4-hydroxy-L-proline ester) [10], poly(α-(4-aminobutyl)-L-glycolic acid) [11], crosslinked poly(aminoester) [12], linear poly(β-aminoester)s [13,14,15,16], polyphosphoester [17,18], polyurethanes [19,20], and polyethylenimine derivatives [21,22]. Among these polymers, linear and network poly(β-aminoester)s also show particular promise as delivery agents, as they are highly biodegradable *in vitro* and easily synthesized via Michael addition of a primary amine to a diacrylate. However, these materials have a significant limitation, namely, low transfection efficiency. One of the primary causes of this poor gene delivery is the inefficient release of polycation/DNA complexes from endosomes into the cytoplasm. There exist three major forms, FR-α, FR-β, and FR-γ, in the folate receptor (FR). Among the forms of FR, FR-α is over expressed in many types of tumor cells. The facts that high affinity FR binding is retained when floate is covalently linked via its γ-carboxy group to a foreign molecule and the prevalence of FR over expression among tumors makes it a good choice for targeting drug delivery to tumors [23]. Polymeric gene vector modified with folic acid was expected to identify specific tissue/cell surface receptors and transfect cells by receptor-mediated endocytosis [24,25,26]. In this study, folate-poly(aminoester) (EPAE-FA) was prepared as an effective gene vector. Folic acid was conjugated to cationic poly(aminoester) to enhance the transfection efficiency for targeting purposes. To evaluate the effect of FA content of EPAE-FA systems on transfection, two EPAE-FA polymers (EPAE-FA12k and EPAE-FA14k) with different FA content were prepared. The effects of FA content of EPAE-FA on transfection ability have been investigated. It was found that a polymer with higher FA content not only has higher transfection efficiency, but also a higher cytotoxicity because of the high cationic charge density.

## 2. Results and Discussion

### 2.1. Structural Characterizations of EPAE-FA

**Scheme 1:**
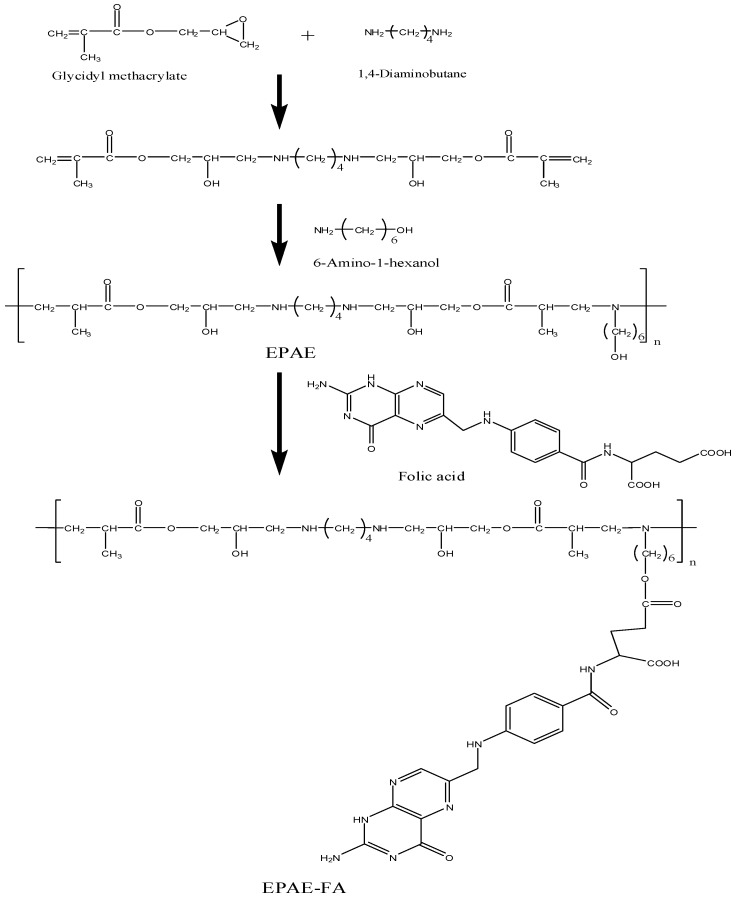
Synthesis of EPAE-FA.

**Figure 1:**
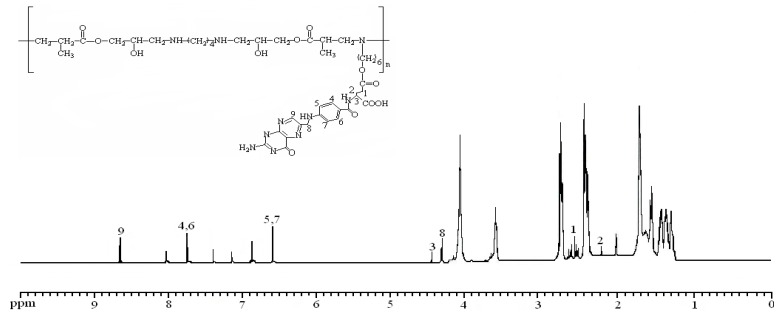
^1^H-NMR spectrum of EPAE-FA.

EPAE-FA was synthesized as shown in Scheme 1. The chemical structure of the polymer was confirmed by FTIR and NMR spectroscopy (Figure 1). The peaks of FT-IR at 1,730 cm^−1^ (C=O, stretching, ester), 1,606 cm^−1^ (N–H, bending, amine), and 3,393 cm^−1^ (O–H, stretching, hydroxyl) represent the characteristic absorptions of EPAE-FA (data not shown). The chemical shifts of characterized protons of EPAE-FA are given in the Experimental section. In addition, the GPC data of EPAE, EPAE-FA12k, and EPAE-FA14k showed that the weight-averaged molecular weights were 8,400 (EPAE), 12,000 (EPAE-FA12k), and 14,100 (EPAE-FA14k) with polydispersities of 1.394, 1.202, and 1.167, respectively, relative to polystyrene standards in THF. The content of folic acid conjugated in EPAE-FA12k and EPAE-FA14k was 19.24% and 39.18%, respectively, obtained on the basis of the calibration curve by using the UV absorbance at 280 nm as the reference. EPAE and EPAE-FA all dissolved in water, DMF, and DMSO.

### 2.2. Size and Zeta-Potential Analysis of Polymer/DNA Complexes

**Figure 2:**
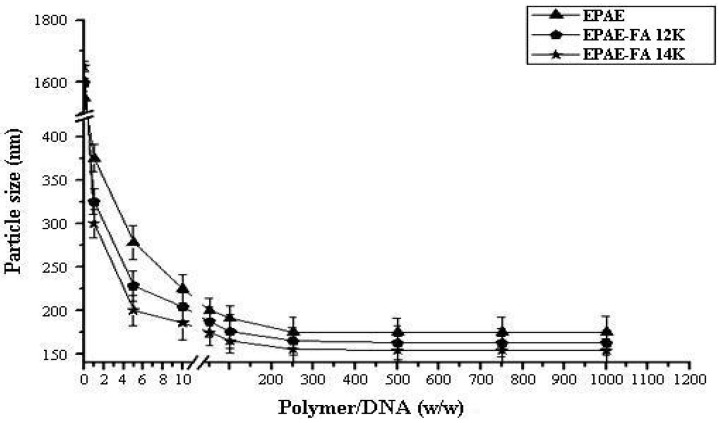
Sizes of EPAE/DNA and EPAE-FAs/DNA complexes prepared at various mass ratios. Results are presented as the mean ± SD (n = 3).

**Figure 3:**
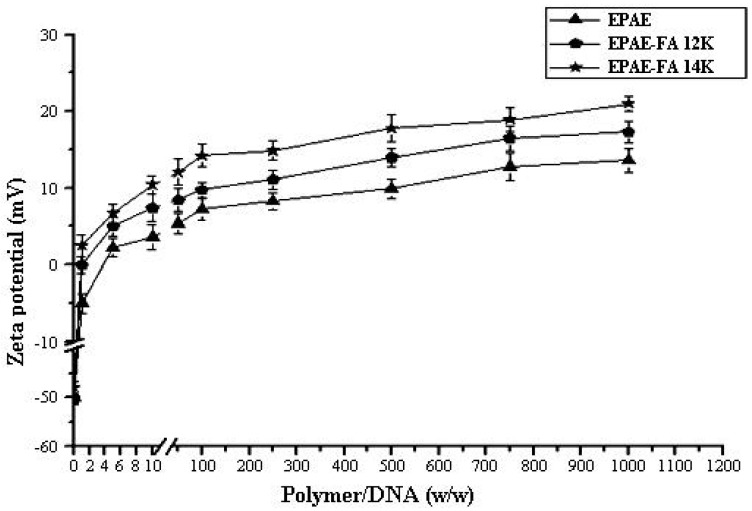
Zeta-potential of EPAE/DNA and EPAE-FAs/DNA complexes prepared at various mass ratios. Results are presented as the mean ± SD (n = 3).

Figure 2 and Figure 3 show the size and Zeta-potential of EPAE/DNA and EPAE-FA/DNA complexes at various mass ratios, determined using dynamic light scattering (DLS) and electrophoretic mobility at 25 °C with Zeta-Potential analysis. The size of complexes decreased as the mass content of the polymers increased until the mass ratio of polymer/DNA reached 250/1. The results show that the average diameters (<200 nm at mass ratio of 70/1) of EPAE/DNA, EPAE-FA12k/DNA, and EPAE-FA14k/DNA fall within the optimal range (40 nm–200 nm) required for cellular endocytosis. The data demonstrates that EPAE, EPAE-FA12k and EPAE-FA14k can condense DNA into nano-complexes. The Zeta-potential of the resulting complex changed from negative to positive charge when the amounts of EPAE and EPAE-FA increased. When the mass ratio of EPAE-FA12k/DNA and EPAE-FA14k/DNA complexes was higher than 2/1, the surface of pCMV-β-gal was fully occupied with the EPAE-FA molecules, forming positive charge complexes. When the mass ratio of EPAE/DNA complexes was higher than 5/1, positive Zeta-potentials of the complexes formed. Complexes with extra positive charges on their surfaces had better interaction with the target cell membrane, resulting in an enhanced uptake.

### 2.3. Buffering Capacity of Polymers

**Figure 4:**
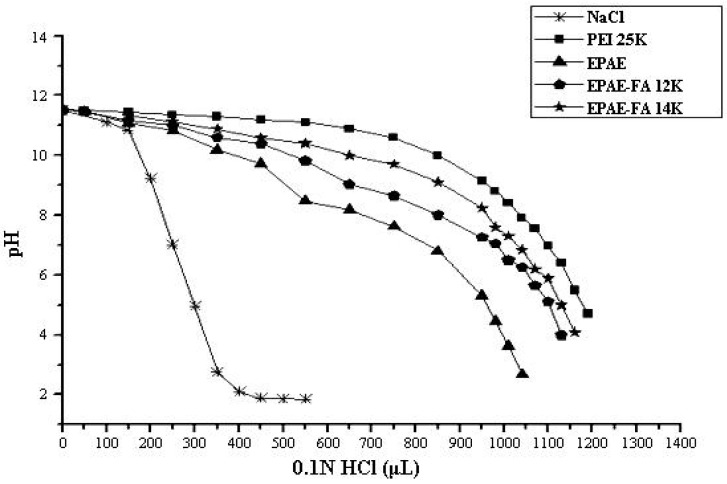
Acid-base titration profile of various polymers with 0.1 N HCl.

Titration studies were performed to determine the buffering capacities of the various polymers regarding a proton buffering effect within the endosomal/lysosomal compartments of the cell (Figure 4). All the polycation solutions had a pH of 11.5 to 11.8 after the addition of 1.0 N NaOH. The non-viral vector PEI showed a buffering capacity over a wide pH range, which is probably due to the high amount of amine functions present in the polymeric chain. The titration protocol of EPAE-FA12k and EPAE-FA14k are similar with that of PEI25k because of the introduction of FA into EPAE. It was also found the quantity of FA can change the buffering region of the polycation. The data show the pH range of EPAE-FA12k to be between 11.8 and 5.4 and that of EPAE to be between 11.8 and 6.6.

### 2.4. Cytotoxicity of Polymers/DNA

**Figure 5:**
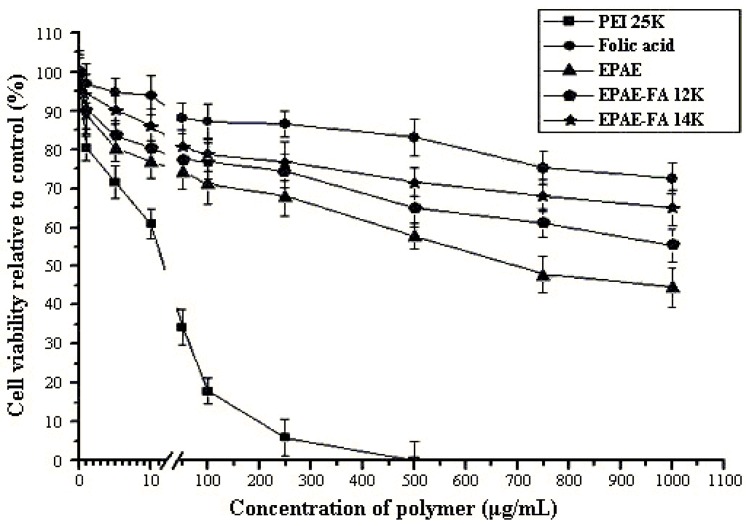
Cytotoxicity of polymer/DNA complexes in COS-7 cells. Results are presented as the mean ± SD (n = 3).

**Figure 6:**
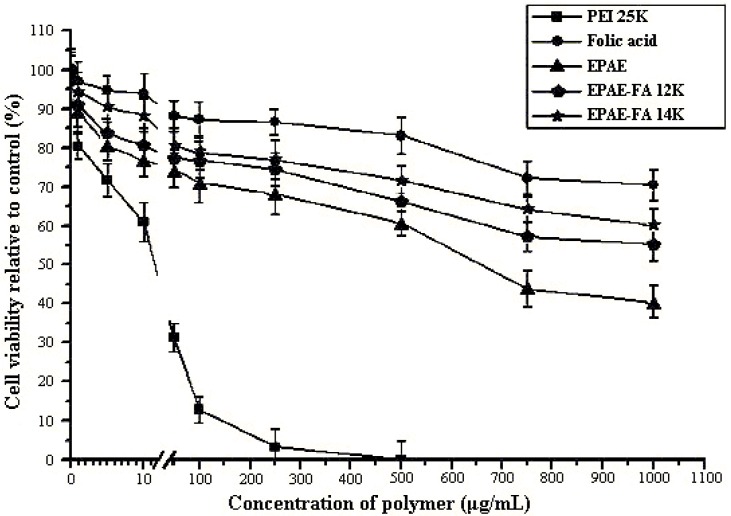
Cytotoxicity of polymer/DNA complexes in HeLa cells. Results are presented as the mean ± SD (n = 3).

A critical element for the gene delivery system is cytotoxicity. Cell damage resulting from a cytotoxic delivery system is deleterious because cells must be capable of supporting translation and transcription. To determine the cytotoxicity of EPAE-FA/DNA for comparison with that of EPAE/DNA and PEI25k/DNA, we performed a XTT assay using the COS-7 and HeLa cell lines. Cells were incubated with increasing amounts of polymers/DNA. Relative cell viabilities of EPAE-FA/DNA, EPAE/DNA, and PEI25k/DNA were shown in Figure 5 and Figure 6. As can be seen, the cell viability of PEI25k/DNA complexes decreased rapidly with an increase in PEI25k concentration, whereas that of EPAE-FA/DNA and EPAE/DNA did not largely change with an increase in EPAE-FA/DNA and EPAE/DNA. The results show that EPAE-FA/DNA and EPAE/DNA exhibited substantially lower toxicity on COS-7 (HeLa) cells than did PEI25k/DNA. We infer that EPAE-FA/DNA and EPAE-FA can provide better cytotoxicity profiles than these presently used for PEI25k/DNA due to the differences in the chemical structure, which reduces the high charge density of the polycation.

### 2.5. DNA Gel Retardation Assay

**Figure 7:**
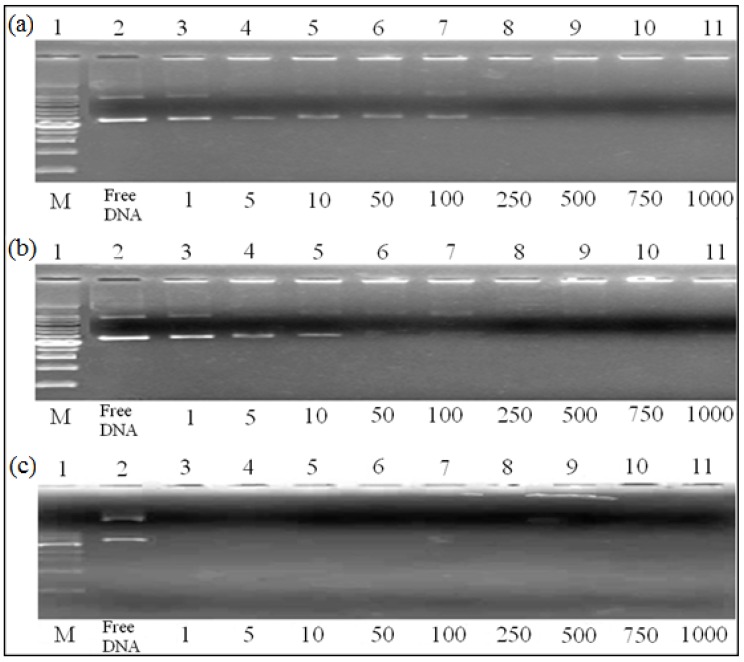
DNA gel retardation assay of EPAE (**a**) EPAE-FA12k; (**b**) and EPAE-FA14k; (**c**) Lanes: (2) 1 μg pCMV-βgal; (3) polymer/pCMV-βgal (w/w): 1/1; (4) polymer/pCMV-βgal(w/w): 5/1; (5) polymer/pCMV-βgal (w/w): 10/1; (6) polymer/pCMV-βgal(w/w): 50/1;(7) polymer/pCMV-βgal (w/w): 100/1; (8) polymer/pCMV-βgal(w/w): 250/1; (9) polymer/pCMV-βgal (w/w): 500/1; (10) polymer/pCMV-βgal (w/w): 750/1; (11) polymer/pCMV-βgal (w/w): 1,000/1.

The electrophoretic mobility behavior of EPAE/DNA, EPAE-FA12k/DNA, and EPAE-14k/DNA is shown in Figure 7(a–c). Increasing amounts of EPAE, EPAE-FA12k, and EPAE-14k/DNA led to the neutralization of DNA negative charges, as shown by gel retardation. The DNA mobility on agarose gel was influenced by the presence of EPAE-FA12k, and EPAE-FA14k.

### 2.6. Cellular Delivery of Plasmid DNA via EPAE-FA Vectors

**Figure 8:**
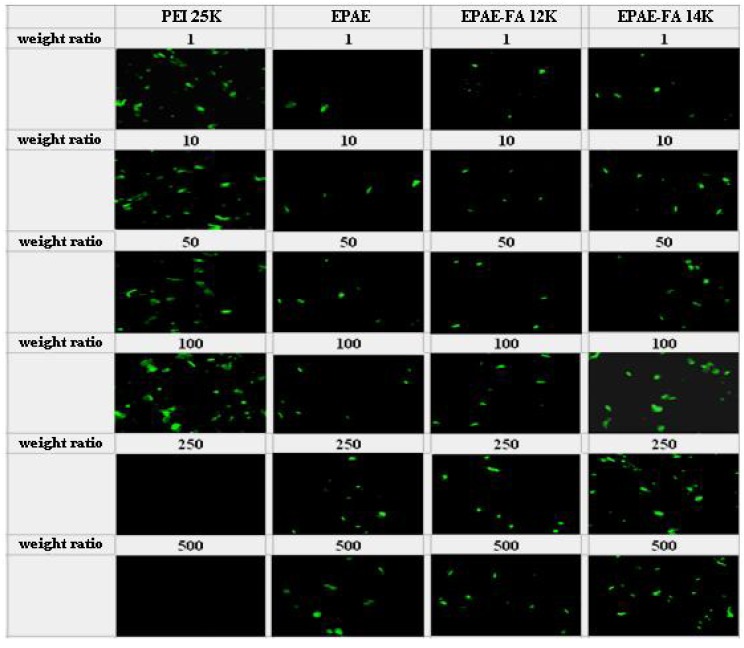
Fluorescence images of GFP expression in COS-7 cells transfected using PEI25, PEI25k, EPAE, EPAE-FA12k, and EPAE-FA14k at various weight ratios.

**Figure 9:**
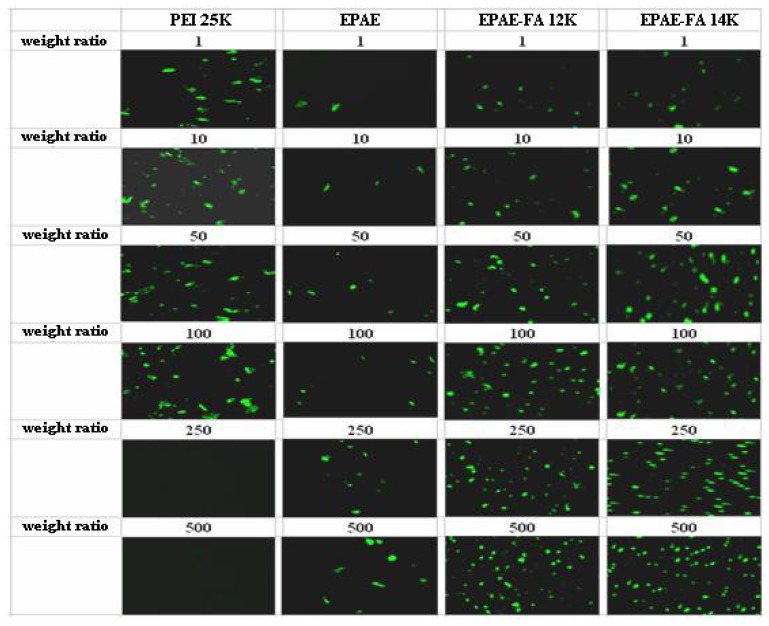
Fluorescence images of GFP expression in HeLa cells transfected using PEI25k, EPAE, EPAE-FA12k, and EPAE-FA14k at various weight ratios.

**Figure 10:**
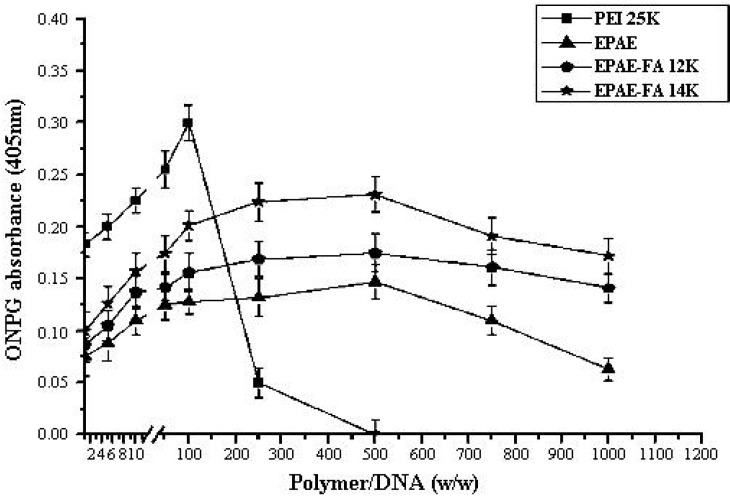
Transfection efficiency of the polymer/DNA complexes into cultured COS-7 cells. Results are presented as the mean ± SD (n = 3).

**Figure 11:**
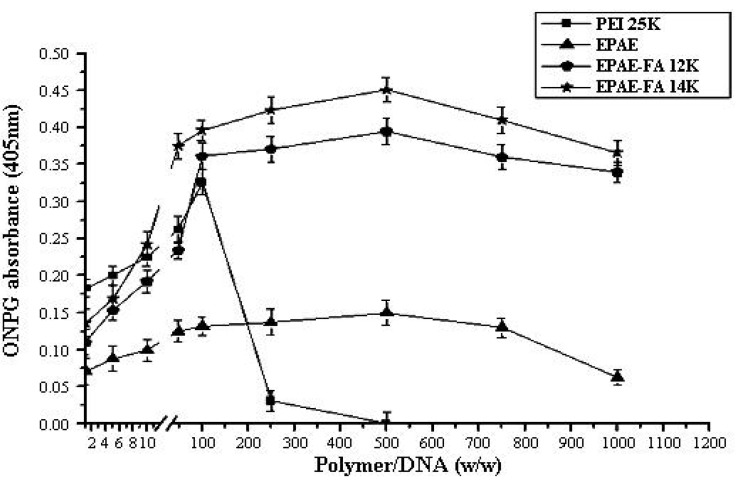
Transfection efficiency of the polymer/DNA complexes into cultured HeLa cells. Results are presented as the mean ± SD (n = 3).

For targeting purposes, the folic acid molecule was grafted to the poly(aminoester)(EPAE). To assess the transfection efficiency mediated by EPAE and EPAE-FA polymers, we performed *in vitro* transfection activity assays using FR-negative (COS-7) and FR-positive (HeLa) cells. The transfection efficiency is studied by fluorescence microscopy (Figure 8 and Figure 9) and the amount of β-galactosidase (equivalent to ONPG absorbance) measured in COS-7 (FR-negative) and HeLa (FR-positive) cells. The images in Figure 8 and Figure 9 show that COS-7 and HeLa cells were transfected with PEI25k/DNA, EPAE/DNA, EPAE-FA12k/DNA, and EPAE-FA14K/DNA complexes, respectively. As shown in Figure 8, for COS-7 cells no obvious difference of fluorescent density was found between EPAE/DNA and EPAE-FA/DNA because COS-7 cell is FR-negative cell. In contrast to COS-7 cells, the fluorescent density showed obvious differences when HeLa cells were transfected with EPAE/DNA and EPAE-FA/DNA complexes (shown in Figure 9). The fluorescent density of HeLa cells was increased when we used EPAE-FA/DNA instead of EPAE/DNA, while there was not much difference in COS-7 cells. These results indicate that the transfection efficiency, mediated by EPAE or EPAE-FA, depended on cell types. EPAE-FA/DNA complexes exhibited higher transfection efficiency than that of EPAE/DNA complexes in FR-positive HeLa cells, whereas no obvious difference in FR-negative COS-7 cells was found. To quantity the transfection efficiency, ONPG was carried out. It was found that the ONPG absorbance of the polymer/DNA complexes were similar to the results of GFP detection (Figure 10 and Figure 11). As can be seen, EPAE-FA14k with higher content of folic acid had the greater transfection efficiency than EPAE-FA12k with lower content of folic acid. It was also found EPAE-FAs had the greater transfection efficiency than EPAE because of the existence of folic acid. The transfection efficiencies of EPAE-FA12k/DNA and EPAE-FA14k/DNA complexes increased with an increase in weight ratios, whereas that of PEI25k/DNA complexes did not observe. The best relative transfection efficiency of EPAE-FA14k/DNA and EPAE-12k/DNA complexes were reached at weight ratio 100/1, which was higher than that of PEI25k/DNA and EPAE/DNA complexes (seen in Figure 11). The EPAE-FA12k/DNA and EPAE-FA14k/DNA complexes were able to transfect HeLa cell *in vitro* with higher transfection efficiency than PEI25k/DNA. This result demonstrated that the EPAE-FA12k/DNA and EPAE-FA14k/DNA complexes were able to transfect FR-positive HeLa cell *in vitro*.

## 3. Experimental

### 3.1. Materials

Glycidyl methacrylate and 1,4-diaminobutane were purchased from Acros Co. (Pittsburgh, PA, USA). N,N-Dimethylethylenediamine and *n*-hexane were obtained from Fluka Co. (St. Gallen, Switzerland). The solvent of N,N-dimethylformamide (DMF, Tedia Co., Fairfield, OH, USA) was dried over calcium hydride and distilled just before use. Folic acid (FA), polyethylenimine (Branched PEI, MW = 25,000), 6-amino-1-hexanol, ethyl isocyanatoacetate, and N-[2-hydroxyethyl]piperazine-N'-[2-ethane-sulfonic acid] (HEPES) were obtained from Sigma Co. (St. Louis, MO, USA). N-methyldibenzopyrazine methyl sulfate (electron-coupling reagent) and sodium (2,3-bis(2-methoxy-4-nitro-5-sulphophenyl)-2H-tetrazolium-5-carboxanilide) (XTT) were purchased from Roche Co. (Buffalo, NY, USA).

### 3.2. Polymer Characterizations

The structures of the polymers were characterized by nuclear magnetic resonance (NMR, AMX-400 spectrometer, Billerica, MA, USA) and Fourier transform infrared (FT-IR, Mattson Galaxy Series 5000 spectroscope, Fremont, CA, USA). The molecular weight and distribution of the polymer was determined by gel permeation chromatography analysis (GPC, Waters Model LC-2410, Milford, MA, USA) based on polystyrene standards in THF.

### 3.3. Synthesis of EPAE-FA12k (EPAE-FA14k)

EPAE was prepared according to the previous report [13]. Folic acid (FA) and EPAE with a –COOH/–OH molar ratio of 1/2 (1/1) were mixed in anhydrous DMSO in a three-necked reaction flask, and then DCC and NHS (equivalent to moles of FA) were added to the reaction mixture. The reaction mixture was magnetically stirred for 24 h at room temperature under N_2_ environment. The insoluble dicyclohexylurea (DCU) was removed by filtration (0.45 μm Teflon filter). Then the filtrate was dialyzed against anhydrous DMSO using a dialysis membrane (MWCO 6000) for further purification. The solution in the dialysis bag was freeze-dried to obtain the product.

### 3.4. Characterization of EPAE Moiety of EPAE-FA

^1^H-NMR (400 MHz, *d*_6_-DMSO, ppm) δ: 1.23~1.35 (16H, –NCH_2_CH_2_CH_2_CH_2_CH_2_CH_2_OH; –CH(CH_3_)CH_2_–; –NHCH_2_CH_2_CH_2_CH_2_NH–), 1.68 (2H, –NCH_2_CH_2_CH_2_CH_2_CH_2_CH_2_OH), 2.12 (2H, –NHCH_2_CH_2_CH_2_CH_2_NH–), 2.41 (2H, –NCH_2_CH_2_CH_2_CH_2_CH_2_CH_2_OH), 2.56 (4H, –NHCH_2_CH_2_CH_2_CH_2_NH–), 2.73 (2H, –OCOCH(CH_3_)–), 2.77(4H, –NHCH_2_CH(OH)–), 3.59 (2H, –NCH_2_CH_2_CH_2_CH_2_CH_2_CH_2_OH), 4.07(2H, –NHCH_2_CH(OH)–), 4.56(4H, –CH(OH)CH_2_OCO–).

### 3.5. Characterization of FA Moiety of EPAE-FA

^1^H-NMR (400MHz, *d*_6_-DMSO, ppm) δ: 2.01(2H, 
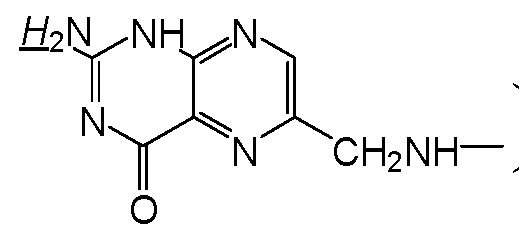
), 2.15 (2H, –OC(O)CH_2_C**H**_2_CH– (COOH)–), 4.29 (2H, –OC(O)C**H**_2_CH_2_CH(COOH)–), 4.52(1H, –OC(O)CH_2_CH_2_C**H**(COOH)–), 6.65 (2H, 
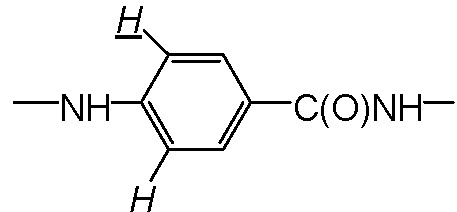
), 7.71 (2H, 
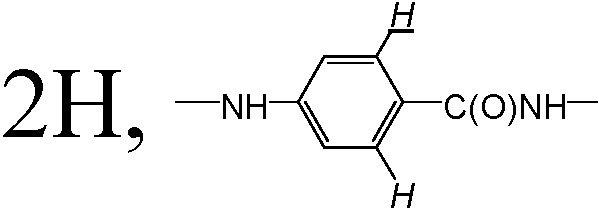
), 8.05 (1H, –CH_2_CH_2_CH(COOH)N**H**-), 8.65(1H, 
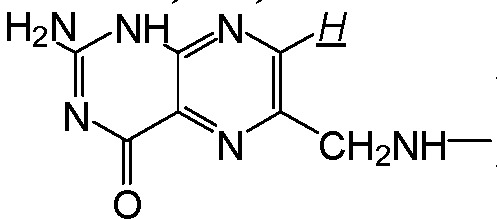
). 

### 3.6. Acid-Base Titration Assay of Polymers

Acid-base titration was used to evaluate the buffering capacity of synthesized cationic polymers. In this assay, polymer (10 mg) was dissolved in 150 mM NaCl (10 mL). 1 N NaOH (100 μL) was then added to the solution to adjust the pH to 11.6 before it was titrated with acid. The solution was titrated with increasing volumes of 0.1 N HCl solutions, and the results were measured using a pH meter.

### 3.7. XTT Assay

The influence of the polymer concentration on the cell viability was evaluated in a cell culture for the various polymers. The cytotoxicities of EPAE-FA/DNA for comparison with that of PEI25k/DNA and EPAE/DNA were evaluated using the XTT assay. In a 96-well plate, COS-7 (HeLa) cells were cultured in complete DMEM and then seeded at a density of 1.0 × 10^4^ cells/well. The cells were incubated at 37 °C and 5% CO_2_ in a humidified atmosphere for 24 h. Subsequently, the cells were incubated for one h in 200 μL FBS-free DMEM containing polymer with various concentrations. The cells were incubated in DMEM as a negative control. After 1 h, the cells were washed with 200 μL PBS solution and replaced by complete DMEM for a further 48 h of incubation. Then, 50 μL of XTT labeling mixture was added to each well and the cells were further incubated at 37 °C for 1 h. Results are expressed as the relative cell viability (%) with respect to control wells containing culture medium.

### 3.8. Formation of Polymer/DNA Complexes

The plasmid pCMV-LacZ (pCMV-βgal) contained a CMV promoter to drive the β-galactosidase (LacZ) gene expression. The plasmid DNA was amplified in *Escherichia* *coli* (DH5α strain) and purified using column chromatography (Qiagen^®^ Plasmid Mega kit, Hilden, Germany). The purified plasmid DNA was dissolved in a tris(hydroxymethyl) methylamine-ethyldiaminetetraacetic acid (Tris-EDTA) buffer (pH 8.0) and determined using the ratio of UV absorbance at 260 nm/280 nm. Monkey SV40 transformed kidney fibroblast COS-7 cells were obtained from American Type Culture Collection (ATCC, CRL-1651). The cells were cultured in Dulbecco’s modified Eagle’s medium (DMEM, GibcoBRL Co., Ltd., San Francisco, CA, USA) supplemented with 10% FBS, 4.5 g/L glucose, 1.5 g/L sodium bicarbonate, and 4 mM L-glutamine, and maintained at 37 °C in a humidified 5% CO_2_-containing atmosphere. Then, 10.0 mg/mL of the polymer was dissolved in 20 mM HEPES buffer (pH 7.4) and its serial dilutions were made with the various N/P ratios of polymers/DNA. The complexes were then allowed to self-assemble in the HEPES buffer. They incubated at room temperature for 30 min before measurements.

### 3.9. Characterizations of Polymer/DNA Complexes

The particle sizes and surface charges of the polymer/DNA complexes were determined by dynamic light scattering (Nicomp 380 system, New York, NY, USA) and electrophoretic mobility at 25 °C with a Zeta-potential system (Nicomp Instrument, New York, NY, USA).

### 3.10. DNA Gel Retardation Assay of Polymer/DNA Complexes

EPAE-FA/DNA complexes and EPAE/DNA complexes were loaded into a 0.7% agarose gel containing ethidium bromide (0.3 g/mL) in a tris-acetate-EDTA (TAE) buffer and performed at 100 V for 45 min. After electrophoresis, the DNA bands were visualized using UV-irradiation. EPAE-FA/DNA complexes and EPAE/DNA complexes with various weight ratios were prepared.

### 3.11. Transfection Protocol and ONPG Assay

COS-7 and HeLa cells were used to evaluate the transfection efficiency of polymer/DNA complexes. The cells were seed in a 96-well plate (1.0 × 10^4^ cells per well) in complete DMEM and incubated for 24 h before transfection trials. The DNA concentration was kept constant at 5 μg/mL (1.0 μg/well) and the amounts of polymers were varied. Two hundred (200) μL solutions of polymer/DNA complexes was taken and incubated with cells for 1 h at 37 °C. The medium was replaced afterwards with complete DMEM and the cells were incubated for another 48 h. For evaluating transfection efficiency, the cells were washed with 0.3 mL PBS and then permeabilized with 20 μL cell lysis buffer at 4 °C for 20 min. An ONPG solution (180 μg/well) was added after lysis treatment and the cells were incubated at 37 °C for 1 h. The expression of pCMV-βgal gene was spectrometrically measured using an ELISA reader at a wavelength of 405 nm.

## 4. Conclusions

Cationic glycidyl-based poly(aminoester)s with folate moiety (EPAE-FA12k and EPAE-FA14k) bearing amino and hydroxyl groups with low cytotoxicity and high transfection efficiency were synthesized and then characterized using FT-IR, NMR, and GPC. In this study, EPAE-FA and DNA had a strong electrostatic interaction to self-assemble nano-particles. The positive charges in the backbone and side chain of EPAE-FA condense DNA to form DNA-polycation complexes. EPAE-FA/DNA complexes displayed comparable or higher transfection efficiency at certain weight ratios in comparison to that of PEI25k/DNA. However, EPAE-FA/DNA complexes showed higher transfection efficiency than those of EPAE/DNA and PEI25k/DNA in FR-positive HeLa cells, whereas no definite difference in FR-negative COS-7 cells was found. Cationic biodegradable EPAE-FA could potentially be used in a non-viral targeting gene delivery system. 
